# Congestion

**Published:** 1881-03

**Authors:** W. W. Hall


### CONGESTION.

BY DR. W. W. HALL.

CONGESTION is the key which unlocks the entire system of medical practice, and makes it a harmonious whole, a system which is beautiful in its simplicity, its efficiency, and truthfulness. Its literal meaning is a pressing together and wedging in, and in this connection applies to the circulation of the blood, which comes out from the heart all bright and sparkling with warmth and life and vigor, entering the largest arteries, which divide and subdivide, getting continually smaller in their branches until they are almost invisible, everywhere depositing those atoms of nutriment with which it was freighted when it left the heart; and, having thus gone to the remotest portions of the body, unloading itself of all its nutriment, each infinitesimal ending artery inosculates, joins on to a corresponding vein quite as small, with this difference; the great aorta as it comes from the heart divides off into lessening branches continually, until they are immensely numerous and small, the volume of blood diminishing all the time, by the amount of nutrient particles it parts with, all full of life. The blood-vessels, on the other hand, called the veins, get larger and fewer, and more full of blood at every inch of their progress; this blood not carrying with it the life of the body, but its death, for it is really the washings out of the system, containing its mere refuse, useless, impure, and dead particles; so loaded is it with these, that it is not sparkling and bright with its cheery and warming red, as in the arteries, but it is dark, lifeless, and dead; finally conveyed in one single channel, one large vein which empties itself directly into the heart, to remain there but a moment, for it is hurried on to the lungs, where, meeting with the pure air drawn into them, it parts with its impurities, hands them over to the air just taken into the lungs, which absorbs them and carries them out in each expiration, and rising upward as it passes the lips and nostrils, it makes its way to the regions above the head, above the houses, above the trees, above the clouds, there to be renovated and made full of life again; again to descend to the earth and enter the nostrils to repeat its purifications and to impart its life, just as all the water of the earth and seas rises to the heavens after use here, and is returned to the earth again in its purity, in the shape of the refreshing showers and of the "beautiful snow."

